# Evaluation of a Low-Cost Strategy for Enumerating CD4 Lymphocyte Absolute Count and Percentage Using the FACSCalibur Flow Cytometer in HIV-Infected Patients from a Resource-Limited Setting

**DOI:** 10.5402/2012/494698

**Published:** 2012-10-23

**Authors:** Gerardo Alvarez-Uria, Raghuprakash Reddy, Srinivasulu Reddy, Praveen K. Naik, Manoranjan Midde

**Affiliations:** ^1^Department of Infectious Diseases, Rural Development Trust Hospital, Kadiri Road, Bathalapalli 515661, India; ^2^Department of Microbiology, Rural Development Trust Hospital, Kadiri Road, Bathalapalli 515661, India

## Abstract

Enumeration of CD4 lymphocytes is essential for the clinical management of HIV-infected patients, but it can be difficult to afford in developing countries. In this study we evaluated a reagent reduction strategy for reducing the cost of enumerating CD4 cell absolute count and percentage using the FACSCalibur flow cytometer (Becton Dickinson). We compared the protocol recommended by the manufacturer with a protocol that used half of the usual amount of CD3/CD4/CD45 monoclonal antibody reagent in 100 samples from HIV-infected patients in a rural hospital in India. The concordance correlation coefficient between the two protocols was 0.976 for CD4 cell count and 0.984 for CD4 cell percentage. We did not find significant bias when performing Deming regression or Bland-Altman analysis. Sensitivity and specificity were 97% and 98.5% for identifying patients with less than 200 CD4 cells/**μ**L, 98.1% and 93.8% for identifying patients with less than 350 CD4 cells/**μ**L, and 100% and 94.7% for identifying patients with less than 25% CD4 cells, respectively. This reagent reduction strategy can be used for reducing the cost of enumerating CD4 lymphocytes in high-volume laboratories from resource-limited settings.

## 1. Introduction

In 2010, it was estimated that 34 million people were living with HIV/AIDS and more than 90% were living in low and middle-income countries [1]. Enumeration of CD4 lymphocytes is essential for the clinical management of HIV-infected people. CD4 lymphocyte count can be used for initiating or stopping prophylaxis against opportunistic infections and for deciding when to initiate antiretroviral therapy against HIV [2]. 

Immunophenotyping by flow cytometer is the most accepted technology for enumeration of CD4 lymphocytes [3]. The FACSCalibur system (Becton Dickinson Biosciences, CA, USA) is a bench-top flow cytometer widely used in laboratories from developed countries and it is considered as the “gold standard” of CD4 counting [4]. However, the high cost of the reagents can be an important limitation for its use in resource-limited settings. In flow cytometry, monoclonal antibodies are the most expensive part of the reagents used to enumerate the absolute count and percentage of CD4 lymphocytes. The objective of this study is to evaluate a reagent reduction strategy for enumerating CD4 lymphocytes with the FACSCalibur system that used half of the usual amount of monoclonal antibodies.

## 2. Methods

The study was performed in the RDT Bathalapalli Hospital, Andhra Pradesh, India. After giving informed consent, peripheral blood was taken from 100 HIV-infected patients attending the Department of Infectious Diseases for enumerating the CD4 lymphocytes as per routine clinical management. 

We compared the protocol recommended by the manufacturer (protocol A) against a protocol that required lesser quantity of reagent (protocol B). Protocol B is an improved version of a protocol suggested by the Application Team of BD in India. Both protocols A and B were performed according to manufacturer's standard operating procedures by trained technicians. Whole blood was collected in a single K3 EDTA tube for each patient and both protocols were performed the same day.

For protocol A, we introduced 20 *μ*L of CD3/CD4/CD45 monoclonal antibody reagent (*BD TriTEST*) and 50 *μ*L of whole blood in a tube with a lyophilized pellet having a calibrated quantity of fluorescent beads (*BD TruCount*). Tubes were incubated for 15 minutes at room temperature (20–25°C) in a dark place before 450 *μ*L of lysing solution was added. The tube was incubated for 15 minutes at room temperature (20–25°C) in a dark place again and then the sample was processed in the FACSCalibur system.

For protocol B, we introduced 10 *μ*L of CD3/CD4/CD45 monoclonal antibody reagent (*BD TriTEST*) and 25 *μ*L of whole blood in a plain polystyrene tube (*BD Falcon*). Tubes were incubated for 15 minutes at room temperature (20–25°C) in a dark place before 450 *μ*L of lysing solution was added. The tube was incubated for 15 minutes at room temperature (20–25°C) in a dark place again before 25 *μ*L of fluorescent bead solution (*BD Liquid Counting Beads*) was added. Then the sample was processed in the FACSCalibur system.

Statistical analysis was performed using Stata Statistical Software (Stata Corporation, Release 11, College Station, TX, USA). Bias was estimated using Deming regression [5]. Deming regression calculates the 95% confidence interval for the estimate of the intercept and the slope of a linear regression equation (*y* = *a*[*y* − intercept] + *b*[slope]*x*). If the value of the *y*-intercept is significantly different from 0 indicates a constant bias and if the value of the slope is significantly different from 1 indicates a proportional bias. The agreement between the two protocols was assessed by using the Altman-Bland method and concordance correlation coefficients (CCCs) [6]. 

## 3. Results and Discussion

The study included 100 samples from HIV-infected patients. The mean CD4 lymphocyte count with protocol A was 366 cells/*μ*L (range 21–1136, standard deviation 254) and the mean CD4 lymphocyte count with protocol B was 349 cells/*μ*L (range 23–1072, standard deviation 248). The mean CD4 lymphocyte percentage with protocol A was 18.4% (range 3–37, standard deviation 8.3) and the mean CD4 lymphocyte percentage with protocol B was 18.4% (range 3–39, standard deviation 8.3). 

**Figure 1:**
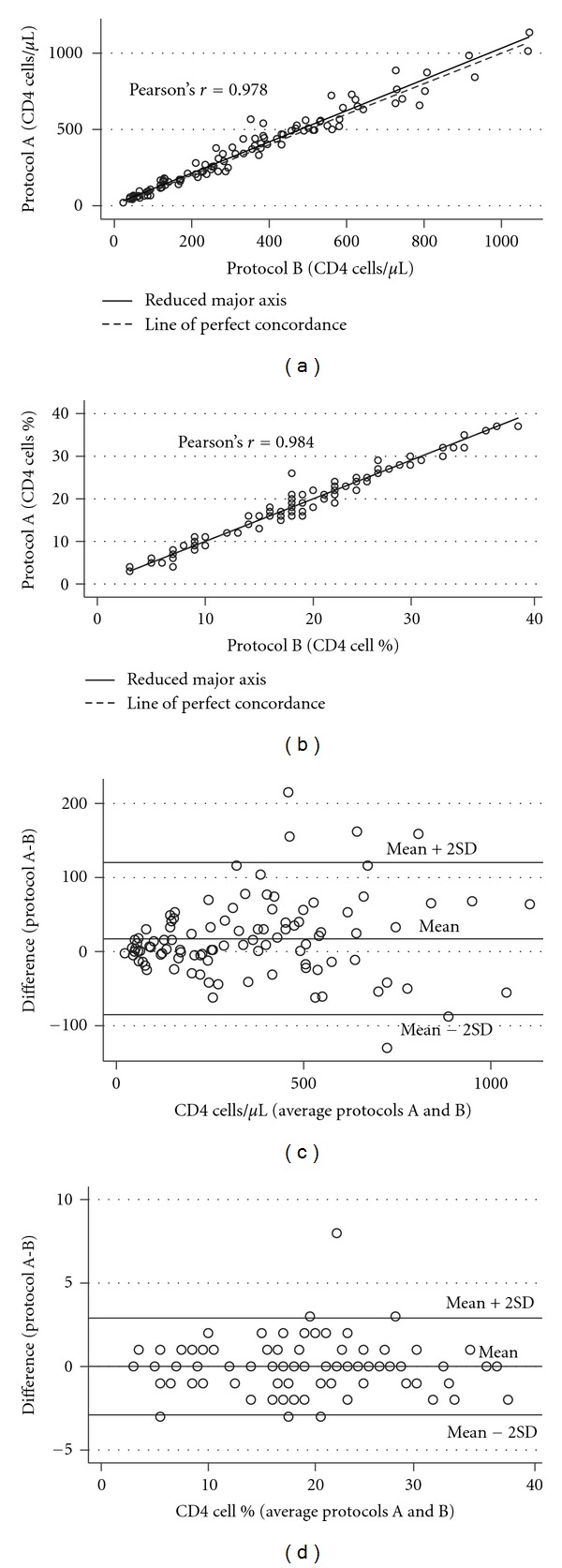
Correlation plots of CD4 cell count (a) and CD4 cell percentage (b), and Bland-Altman plots of CD4 cell count (c) and CD4 cell percentage (d) of protocol A versus protocol B.

Estimation of bias and agreement between the two protocols are presented in Table 1. When performing Deming regression, we did not find any significant bias for CD4 cell count or CD4 cell percentage as the confidence intervals for *y*-intercepts included 0 and the confidence intervals for slopes included 1. The concordance correlation coefficient was slightly higher for CD4 cell percentage than for CD4 cell count. Correlation and Bland-Altman plots are shown in Figure 1. Bland-Altman analysis showed close agreement between the two protocols. 

Sensitivities and specificities of protocol B for three clinically relevant cutoffs are presented in Table 2. Patients having less than 200 CD4 cells/*μ*L need to initiate prophylaxis against *Pneumocystis jirovecii* pneumonia and this cutoff is useful for identifying patients in risk of opportunistic infections [2]. In HIV-infected patients, having less than 350 CD4 cells/*μ*L is an indication for initiating antiretroviral therapy in patients older than 5 years and having less than 25% CD4 lymphocytes is an indication for initiating antiretroviral therapy in children aged 2 to 5 years [2, 7].

Although the reagent reduction strategy has been successfully evaluated in previous studies [8], to our knowledge this is the first time that this strategy has been evaluated for the FACSCalibur system. The cost of FACSCalibur reagents is volume dependent but ranges from 3 to 7 United States dollars (USD) per test [4]. Using protocol B, the cost of the reagents can be reduced by a half, making the final cost per test lower than other low-cost technologies [4]. The FACSCalibur system can be especially useful for laboratories receiving high volume of samples because it is a single platform system, does not require highly trained operators, and provides a convenient walk-away automation through a sample loader [4, 9]. Although this cost reduction makes the FACSCalibur system a very interesting option for high volume laboratories, the cost of the instrument is about 75,000 USD [4], which is considerably higher than other technologies, so the system may not be feasible for laboratories receiving a low volume of samples in resource-limited settings.

## 4. Conclusions

Protocol B is able to provide reliable results of CD4 cell count and percentage with half of usual amount of monoclonal antibody reagent. This reagent reduction strategy can be used for reducing the cost of enumerating CD4 lymphocytes with the FACSCalibur system.

## Figures and Tables

**Table 1:** Concordance correlation coefficients, bias estimation by Deming regression and Bland-Altman analysis between protocol A and B.

	CCC (95% CI)	*y*-intercept (95% CI)	Slope (95% CI)	Mean difference (95% LOA)
CD4 cell count	0.976 (0.966 to 0.985)	8.68 (−5.18 to 22.54)	1.02 (0.97 to 1.08)	17.18 (−85.88 to 120.24)
CD4 cell percentage	0.984 (0.978 to 0.99)	0.03 (−0.53 to 0.59)	1 (0.97 to 1.03)	0.01 (−2.91 to 2.93)

CCC: concordance correlation coefficient; CI: confidence interval; LOA: limits of agreement.

**Table 2:** Sensitivity and specificity with Wilson's 95% confidence intervals of protocol B to identify patients having <200 cells/*μ*L, <350 cells/*μ*L, and <25% CD4 cells with protocol A.

	Sensitivity % (95% CI)	Specificity % (95% CI)
<200 cells/*μ*L	97 (84.7–99.5)	98.5 (92–99.7)
<350 cells/*μ*L	98.1 (89.9–99.7)	93.8 (83.2–97.9)
<25% CD4 cells	100 (95.5–100)	94.7 (75.4–99.1)

CI: confidence interval.
